# Gys1 Antisense Therapy Prevents Disease-Driving Aggregates and Epileptiform Discharges in a Lafora Disease Mouse Model

**DOI:** 10.1007/s13311-023-01434-9

**Published:** 2023-09-12

**Authors:** Katherine J. Donohue, Bethany Fitzsimmons, Ronald C. Bruntz, Kia H. Markussen, Lyndsay E. A. Young, Harrison A. Clarke, Peyton T. Coburn, Laiken E. Griffith, William Sanders, Jack Klier, Sara N. Burke, Andrew P. Maurer, Berge A. Minassian, Ramon C. Sun, Holly B. Kordasiewisz, Matthew S. Gentry

**Affiliations:** 1 Department of Molecular and Cellular Biochemistry, University of Kentucky, Lexington, KY 40506, USA; 2 Department of Antisense Drug Discovery, Ionis Pharmaceuticals, Carlsbad, CA 92010 USA; 3 Department of Neuroscience and Center for Cognitive Aging and Memory, University of Florida, Gainesville, FL 32610 USA; 4 Division of Neurology, Department of Pediatrics, University of Texas Southwestern Medical Center, Dallas, TX 75390 USA; 5 Department of Biochemistry and Molecular Biology, University of Florida, Gainesville, FL 32610 USA

**Keywords:** Lafora disease, Antisense oligonucleotide, Glycogen, Glycogen synthase, Glycogen storage disease

## Abstract

Patients with Lafora disease have a mutation in *EPM2A* or *EPM2B*, resulting in dysregulation of glycogen metabolism throughout the body and aberrant glycogen molecules that aggregate into Lafora bodies. Lafora bodies are particularly damaging in the brain, where the aggregation drives seizures with increasing severity and frequency, coupled with neurodegeneration. Previous work employed mouse genetic models to reduce glycogen synthesis by approximately 50%, and this strategy significantly reduced Lafora body formation and disease phenotypes. Therefore, an antisense oligonucleotide (ASO) was developed to reduce glycogen synthesis in the brain by targeting glycogen synthase 1 (*Gys1*). To test the distribution and efficacy of this drug, the Gys1-ASO was administered to *Epm2b-/-* mice via intracerebroventricular administration at 4, 7, and 10 months. The mice were then sacrificed at 13 months and their brains analyzed for Gys1 expression, glycogen aggregation, and neuronal excitability. The mice treated with Gys1-ASO exhibited decreased Gys1 protein levels, decreased glycogen aggregation, and reduced epileptiform discharges compared to untreated *Epm2b-/-* mice. This work provides proof of concept that a Gys1-ASO halts disease progression of *EPM2B* mutations of Lafora disease.

**Supplementary Information:**

The online version contains supplementary material available at 10.1007/s13311-023-01434-9.

## Introduction

Lafora disease (LD) is a devastating childhood-onset epilepsy and dementia, brought on by autosomal recessive mutations in either *EPM2A* or *EPM2B*, which encode for the glycogen phosphatase laforin and E3 ubiquitin ligase malin, respectively [1–8]. These two enzymes regulate glycogen storage in tissues throughout the body. When the function of either protein is impaired, aberrant glycogen with hyperphosphorylation and extended glucose chains form and aggregate into Lafora bodies (LBs) [9–14]. Patients with LD develop seemingly normal until early adolescence, when they experience seizures with increasing frequency and severity, coupled with neuroinflammation and neurodegeneration that is accompanied by rapid childhood dementia [15–17]. There is currently no approved therapy for LD, and the progression of the disease proves fatal after approximately 11 years from the onset of symptoms [18, 19].

Among LD patients, about half exhibit a mutation in *EPM2A*, while the other half present with a mutation in *EPM2B* [19]. Therefore, an ideal therapeutic should demonstrate efficacy in LD patients regardless of which gene is mutated. While the precise mechanism of the disease is not fully understood, particularly in regard to the contribution of malin ubiquitination, the loss of malin or laforin function yields aberrant glycogen and subsequent LB aggregation that drives very similar disease progression in patients with mutations in either gene. Multiple studies have demonstrated that LBs are the driving agent behind disease progression [20–26]. Multiple laboratories hypothesized that reducing glycogen synthesis would slow LB aggregation and therefore slow disease progression. Subsequent studies in multiple murine models from numerous laboratories demonstrated that when glycogen synthesis is reduced by approximately 50% in LD mice, LB accumulation is significantly reduced, and disease symptoms are reduced or abrogated [21–23, 25]. Therefore, glycogen synthase 1 (GYS1), the enzyme that drives glycogen synthesis in the brain, was selected as a target for LD therapeutics.

A *Gys1* antisense oligonucleotide (ASO) was recently developed that decreased Gys1 expression in the brain for LD mouse models [27]. The efficacy of the Gys1-ASO treatment was tested in young *Epm2a-/-*, laforin knockout (KO), LD mice compared to treatment efficacy of older mice and with varied length of treatment [27]. Treatment was more effective the earlier the Gys1-ASO was administered. Although Gys1-ASO treatment did not clear existing LBs, the Gys1-ASO did halt LB formation and reduce neuroinflammation in laforin KO mice [27].

Given the promising results in long-term administration to laforin KO mice, we sought to determine if the Gys1-ASO therapy produced similar results in the *Epm2b-/-,* malin KO, mouse model and to define if the Gys1-ASO modulated neuronal excitability. Therefore, we treated a cohort of malin KO mice with the same Gys1-ASO and assessed the impact of treatment on Gys1 expression and glycogen accumulation throughout the brain in each treatment cohort. Using histological and mass spectrometry analyses, Gys1-ASO treatment significantly reduced the expression of Gys1 and synthesis of glycogen in the brain, reducing glycogen accumulation in LBs. Furthermore, brain slices from the malin KO mice were analyzed for neuronal excitability, and slices treated with Gys1-ASO exhibited a reduction to near WT levels of excitability, suggesting that treatment with the Gys1-ASO could decrease the neuronal hyperexcitability observed in LD. Cumulatively, our analyses, coupled with previous results, demonstrate critical proof of concept data that this Gys1-ASO is effective for LD caused by mutations in *Epm2a* or *Epm2b*.

## Materials and Methods

### Mice and ASO Delivery

The malin KO mouse model and injection protocol were described previously. All animal procedures were approved by Ionis Pharmaceuticals Institutional Animal Care and Use Committees [27, 28]. In brief, male and female mice were anesthetized with isoflurane, and 300-µg ASO in 10-µL PBS was injected intracerebroventrically (ICV) at 4, 7, and 10 months. Stereotactic injection coordinates were 0.3 mm anterior/posterior (anterior to bregma), 1.0 mm to right or left medial/lateral, and − 3.0 mm dorsal/ventral. The mouse-specific Gys1-ASO sequence is 5′-CATGCTTCATTTCTTTATTG-3′. Littermate controls received injections of a non-target ASO, 5′-CCTATAGGACTATCCAGGAA-3′ (control ASO), or PBS. Each treatment group began with 5 male and 5 female mice, although 2 female mice in the control ASO group died before the study completion. Mice were sacrificed at 13 months by cervical dislocation and decapitation. The hemisphere ipsilateral to the injection site was snap-frozen in liquid nitrogen for qRT-PCR and biochemical analyses, and the other was immersed in 10% neutral buffered formalin for histo- and immunohisto-pathology and matrix-assisted laser desorption/ionization (MALDI) analysis.

### mRNA Expression Analysis of Gys1 and Inflammation Markers

Sample RNA was extracted utilizing the Qiagen RNeasy Mini Kit. Prior to usage, samples were homogenized with a Qiagen QIAshredder. Homogenized tissue was centrifuged at full speed for 3 min, and the supernatant was added to RNeasy columns for subsequent steps. DNase digestion was performed with the RNase-Free PureLink DNase set (Invitrogen). mRNA expression was measured using qRT-PCR with Reliance One-Step Multiplex RT-qPCR Supermix (Bio-Rad) for cDNA synthesis and Bio-Rad PrimePCR probes for Gys1 (assay id: qHsaCIP0032887), CCL5 (assay id: qMmuCEP0057452), CXCL10 (assay id: qMmuCEP0057926), MMP3 (assay id: qMmuCEP0057596), and GAPDH (assay id: qMmuCEP0039581).

### Gys1 Protein Expression Analysis

Three male and three female mice were randomly selected from each treatment group for analysis of Gys1 protein expression via Western blot. Frozen brain tissue was homogenized using a SPEX Sample Prep Freezer/mill 6775 and placed into RIPA buffer (50 mM Tris–HCl, 2% SDS, pH 7.4) containing protease and phosphatase inhibitors (Sigma-Aldrich 5872S). Protein concentration was determined using a BCA Protein Assay Kit (Thermo Scientific 23,225). Protein was standardized at 30 µg and diluted with 6 × SDS loading buffer. Samples were boiled (96 °C, 5 min) and loaded onto a 4–15% stain-free TGX gel (Bio-Rad #4,568,085) for Western blotting. A wet transfer was performed overnight at 10 V to ensure the transfer of protein onto the PVDF membrane (Bio-Rad). Anti-GYS1 (Cell Signaling #3893) and anti-beta actin (Bio-Rad #12,004,163) were utilized as primary antibodies, and goat anti-rabbit HRP-conjugated secondary antibody (Bio-Rad #1,706,515) was utilized and developed with Clarity ECL Substrate (Bio-Rad #1,705,060). After developing, blots were analyzed using a ChemiDoc Imaging System (Bio-Rad #27,444). To quantify the blots, blots were analyzed using ImageJ software with the rectangle and integration tools to measure the intensity of each band. Data were exported and graphed in GraphPad Prism and analyzed using a one-way ANOVA.

### Histological Analysis

Formalin-fixed brains were embedded in paraffin blocks. Samples were sectioned, placed on slides, and stained with periodic acid-Schiff (PAS) or IV58B6 anti-glycogen antibody [29]. Slides were scanned using a Zeiss Axio Scan Z1 and then loaded into HALO v3.3.2541.345 software designed by Indica Labs. HALO software was used to annotate brain regions including the hippocampus and the cerebellum; then, Indica Labs Area Quantification v1.0 was used to quantify the percent positive stain. Four male and four female mouse brain slices with comparable depth of slice through the hippocampus were analyzed for each treatment group. Results were graphed in GraphPad Prism and analyzed using an ordinary one-way ANOVA with the Tukey test for multiple comparisons between treatment groups.

### Glycogen Quantification via MALDI Analysis

Tissues were sectioned at 4 μm and mounted on positively charged glass slides for MALDI imaging with 3 brains represented on each slide according to a previously described protocol [30]. Slides were prepared for MALDI analysis using a previously described method [31, 32]. In brief, the slides were heated at 60 °C for 1 h, allowed to cool, and then deparaffinized by washing twice in xylene (3 min each). Tissue sections were rehydrated by submerging the slide twice in 100% ethanol (1 min each), once in 95% ethanol (1 min), once in 70% ethanol (1 min), and twice in water (3 min each). After the water wash, each slide was transferred to a coplin mailer containing the citraconic anhydride buffer for antigen retrieval, and the mailer was placed in a vegetable steamer for 25 min. Citraconic anhydride (Thermo) buffer was prepared by adding 25-mL citraconic anhydride in 50-mL water and adjusted to pH 3 with HCl. After cooling, the buffer was exchanged with water five times. The slide was then desiccated prior to enzymatic digestion. An HTX spray station (HTX) was used to coat the slide with a 0.2-mL aqueous solution of isoamylase (4 units/slide). The spray nozzle was heated to 45 °C, and the spray velocity was 900 m/min. Slides were then incubated at 37 °C for 2 h in a humidified chamber and dried in a desiccator prior to matrix application (a-cyano-4-hydroxycinnamic acid matrix (0.021-g CHCA in 3-mL 50% acetonitrile/50% water and 12-mL TFA) applied with HTX sprayer). For the detection of glycogen, a Waters SynaptG2-Xs high-definition mass spectrometer equipped with traveling wave ion mobility was used. The laser was operated at 1000 Hz with an energy of 200 AU and spot size of 50 mm, and mass range is set at 500–3000 m/z. Images of glycogen were generated using the waters HDI software [30].

### Measurement of Neuronal Excitability

Malin KO mice and wild-type littermates at 4 months of age were selected and divided into treatment groups: ICV vehicle-injected wild-type mice, ICV vehicle-injected MKO mice, and ICV Gys1-ASO-injected Epm2b-/- mice, with *n* = 10 (5 males and 5 females). Mice were injected with vehicle or ASO as described previously [27]. Three months post-injection, the MKO mice and wild-type littermates were assessed using multiple-electrode analysis (MEA) at NeuroService Alliance. Mice were sacrificed by fast decapitation and the brains soaked in ice-cold oxygenated buffer (2-mM KCl, 7-mM MgCl_2_, 1.2-mM NaH_2_PO_4_, 0.5-mM CaCl_2_, 25-mM NaHCO_3_, 11-mM glucose, and 250-mM sucrose). Slices from the hippocampus were cut in the horizontal plane with a Leica VT1200S vibratome. Slices recovered at 32 °C for 60 min in artificial cerebrospinal fluid (aCSF) (125-mM NaCl, 3.5-mM KCl, 1.2-mM NaH_2_PO_4_, 2-mM CaCl_2_, 25-mM NaHCO_3_, and 11-mM glucose). Throughout the experiment, slices were continuously perfused with oxygenated (95% O_2_/5% CO_2_) aCSF continuously heated at 37 °C. Fifteen contralateral hippocampal slices from each treatment group were placed on the MEA (Multichannel Systems GmbH, Reutlingen, Germany), and spontaneous firing (SF) activity and possible occurrences of epileptiform discharge (ED) were monitored for 60 min after recovery in three different buffers designed to increase neuronal excitability. For the first 20 min, slices were treated with 3.5-mM K^+^, during the next 20 min, potassium ion concentration was increased to 7 mM to enhance firing activity, and during the final 20 min, 100-nM kainic acid was administered; the recording was terminated with an injection of 1-μM tetrodotoxin (TTX), a sodium channel blocker inhibiting action potential firing, to validate the recording by ceasing all neuronal activity.

For the spontaneous firing (SF) rate and epileptiform discharge (ED) event analyses, only channels that had confirmed action potentials were included in the calculation of mean values for each mouse. Several mice in each experimental group did not have usable data based on a failure to obtain action potentials for recording validation and were not included in the analyses. For the SF analysis, raw data of neuron firing was filtered with a high pass filter at 200 Hz, and the firing rate detected at each electrode was averaged over 30-s time slots from the final 5 min of each treatment period to avoid any potential residual signal from the previous brain slice buffer conditions. The recordings revealed that the slices from all treatment conditions exhibited maximal neuronal excitability during the 7.0-mM K^+^ concentration period, resulting in no further increase of SF after KA was administered. Therefore, the baseline 3.5-mM K^+^ 20-min control period was utilized for data analysis of ED events between treatment groups. For ED analysis, raw data were filtered with a low pass filter set at 20 Hz. Detected epileptiform events required an amplitude greater than 15 μV followed by 200 ms of dead time in order to be counted as an ED. The ED frequency was averaged in 1-min time slots for validated electrodes.

### Statistical Analyses

All data points are represented with the mean ± SD, with the exception of the electrophysiology data and PAS histogram analyses, which are represented with the mean ± SEM. GraphPad Prism 9.3.1 was used to perform all statistical analyses. An unpaired *t*-test or an ordinary one-way ANOVA with the Tukey test for multiple comparisons was used to assess significance as indicated in each figure. For electrophysiology data analyses, the SF and ED frequency was averaged across slices and channels from the same mouse and pooled for mice in the same treatment group. Thus, for all statistical analyses, sample sizes were based on number of mice (that is, biological replicates) and not number of technical replicates (that is, slices or recording sites). A mixed effects ANOVA was used to compare SF between experimental groups and recording conditions with recording condition as the repeated measure and group (WT vehicle, MKO vehicle and MKO Gys1 ASO) as the independent factor. A Greenhouse–Geisser correction was implemented for unequal variances, and post hoc analyses were subject to Tukey’s multiple comparison tests. The same analysis procedures were used for the ED analysis, with the exception that time was the within subject variable. Significant levels are indicated by asterisks: *, *P* < 0.05; **, *P* < 0.01; and ***, *P* < 0.001.

## Results

### Gys1-ASO Decreases Gys1 mRNA and Gys1 Protein Expression

**Fig. 1:**
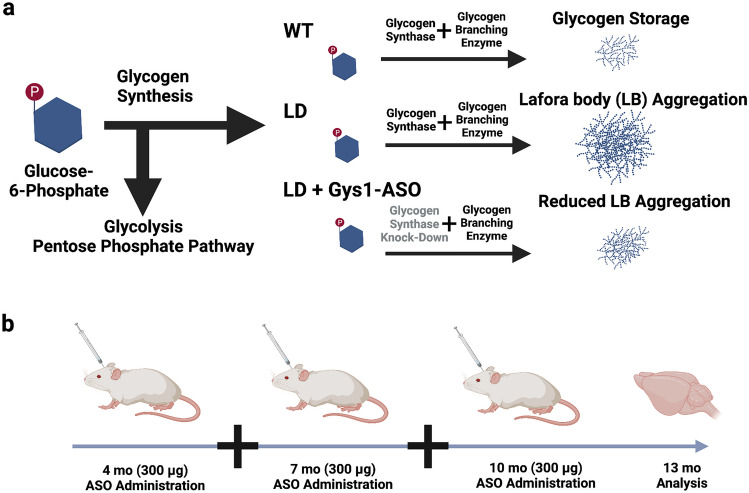
Antisense oligonucleotide (ASO) administration to halt Lafora body formation. **a** Schematic depicting the fate of glucose in Lafora disease (LD) compared to wild-type (WT) and the predicted impact of Gys1-ASO administration. **b** Schematic displaying the timeline for Gys1-ASO administration and testing in WT and LD mouse model. Created with BioRender.com

As previously demonstrated, a Gys1-ASO was developed to knockdown glycogen synthase expression in the brain and was validated as a therapeutic agent in laforin KO mice [27] (Fig. 1a). To test the Gys1-ASO as a therapeutic approach in malin KO mice, we assigned male and female mice into groups (*n* = 5 per group) for no surgery or surgery with vehicle, control scrambled ASO, or Gys1-ASO via intracerebroventricular (ICV) injections. Mice were administered treatment at 4, 7, and 10 months of age and were sacrificed at 13 months (Fig. 1b). Brain hemispheres were divided via sagittal dissection with the side ipsilateral to the ICV injection flash frozen and the side contralateral to the ICV injection fixed in formalin and embedded in paraffin for analysis.

**Fig. 2:**
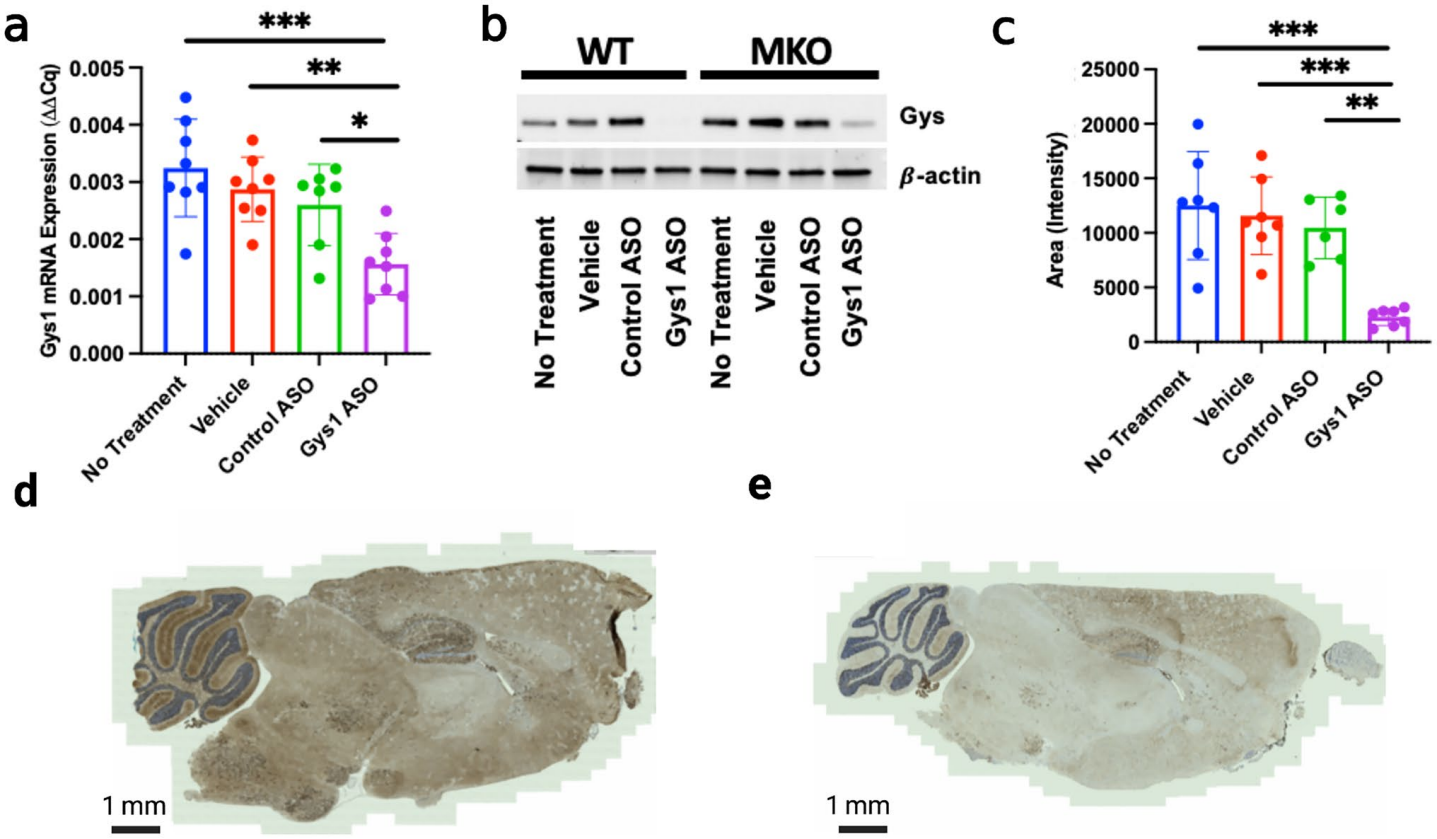
Gys1-ASO knockdown of brain glycogen synthase (*Gys1*). **a** mRNA transcription levels of *Gys1* for the *Epm2b-/-*, malin knockout (MKO), and mouse cohort. **b** Representative blot of Gys1 protein expression in MKO mice, additional blots included in Sup. Fig. 1. **c** Quantitation of Western blots for Gys1 protein expression in MKO mice. **d** Representative IHC stain for Gys1 in control ASO-treated MKO mouse. **e** Representative IHC stain for Gys1 in Gys1-ASO-treated MKO mouse. Statistical significance was calculated using an ordinary one-way ANOVA with post hoc Tukey test for multiple comparisons, where *P < 0.05, **P < 0.01, and ***P < 0.001

To evaluate the efficacy of the Gys1-ASO, levels of *Gys1* mRNA and protein expression were analyzed. The frozen brain tissue was pulverized, cells were lysed, RNA was extracted from the lysate, and qRT-PCR was performed to quantify mRNA expression of *Gys1*. *Gys1* mRNA decreased significantly in the Gys1-ASO-treated cohort (Fig. 2a). The lysate was then immunoblotted to assess Gys1 protein expression. Gys1-ASO treatment dramatically decreased Gys1 protein levels compared to control animals (Fig. 2b and c, Sup. Fig. 1). To assess spatial Gys1 reduction, anti-GYS1 immunohistochemistry (IHC) was performed and revealed a striking decrease in Gys1 levels in all brain regions for mice treated with the Gys1-ASO (Fig. 2d and e). Additionally, mRNA expression levels for CCL5, CLCX10, and MPP3, three common neuroinflammation markers, were quantified from the brain tissue lysate using qRT-PCR to assess the impact of ASO administration on neuroinflammation. As expected, the MKO mice exhibited higher levels of neuroinflammation compared to the WT mice [33]. There was not a significant change in neuroinflammation between treatment groups, likely due to treatment timing (Sup. Fig. 2a–c).

### Gys1-ASO Treatment Reduces Glycogen Accumulation

**Fig. 3:**
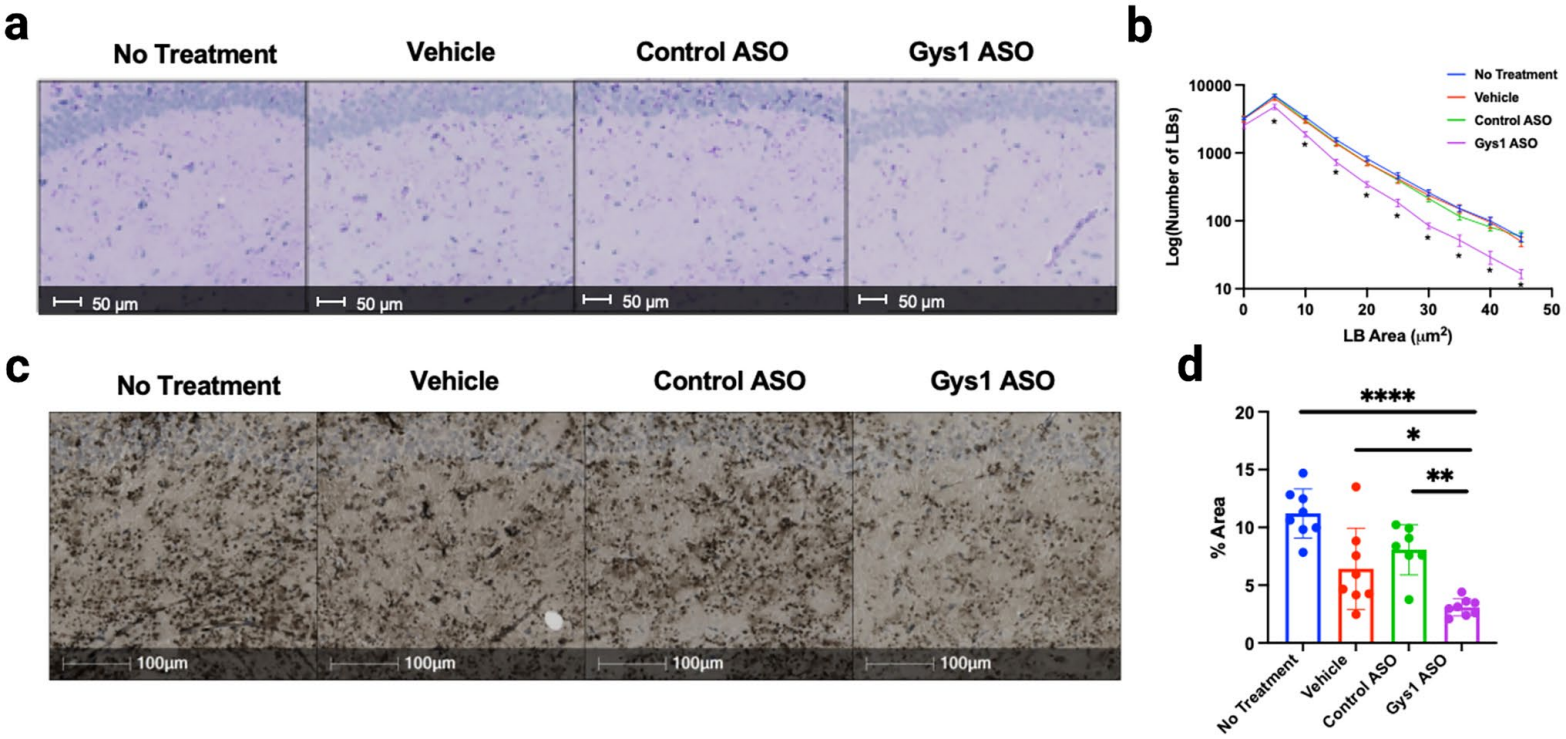
Lafora body aggregation in the hippocampus. **a** Representative images of PAS-stained hippocampi in MKO mice. **b** Quantitation of percent area of Lafora bodies in PAS-stained hippocampi of MKO mice. **c** Representative images of IHC using the IV58B6 α-glycogen antibody to stain hippocampi of MKO mice. **d** Percent area of hippocampal staining of MKO mice with the IV58B6 antibody. Statistical significance was calculated using one-way ANOVA with post hoc Tukey test for multiple comparison, where *P < 0.05, **P < 0.01, and ****P < 0.0001

Historically, periodic acid-Schiff (PAS) staining was the primary method used to assess LB accumulation. PAS staining targets carbohydrate macromolecules, like glycogen and LBs, by using periodic acid to break their carbon–carbon bonds and oxidize the hydroxyl groups to produce two aldehyde groups, and then, Schiff reagent reacts with the aldehyde group to produce a fuchsia or deep purple staining [34]. In LD murine models, the hippocampus exhibits significant accumulation of LBs via PAS staining. Therefore, hippocampal regions from each of the malin KO treatment groups were analyzed by PAS staining [4, 35] (Fig. 3a). HALO software was used to quantify the number of LBs in the hippocampus and plot the number of LBs in each size bracket. We observed a significant decrease in the number of larger LB aggregates (area > 5 μm^2^) in the Gys1-ASO treatment group compared to the controls (Fig. 3b).

While PAS staining allows comparison to previous studies, the IV58B6 anti-glycogen antibody exhibits increased specificity for glycogen and decreased variability [29]. We performed IHC using IV58B6 to assess glycogen accumulation in each malin KO treatment group (Fig. 3c). IHC of IV58B6 staining in the hippocampus was significantly lower in the Gys1-ASO-treated group compared to all the control groups (Fig. 3d).

**Fig. 4:**
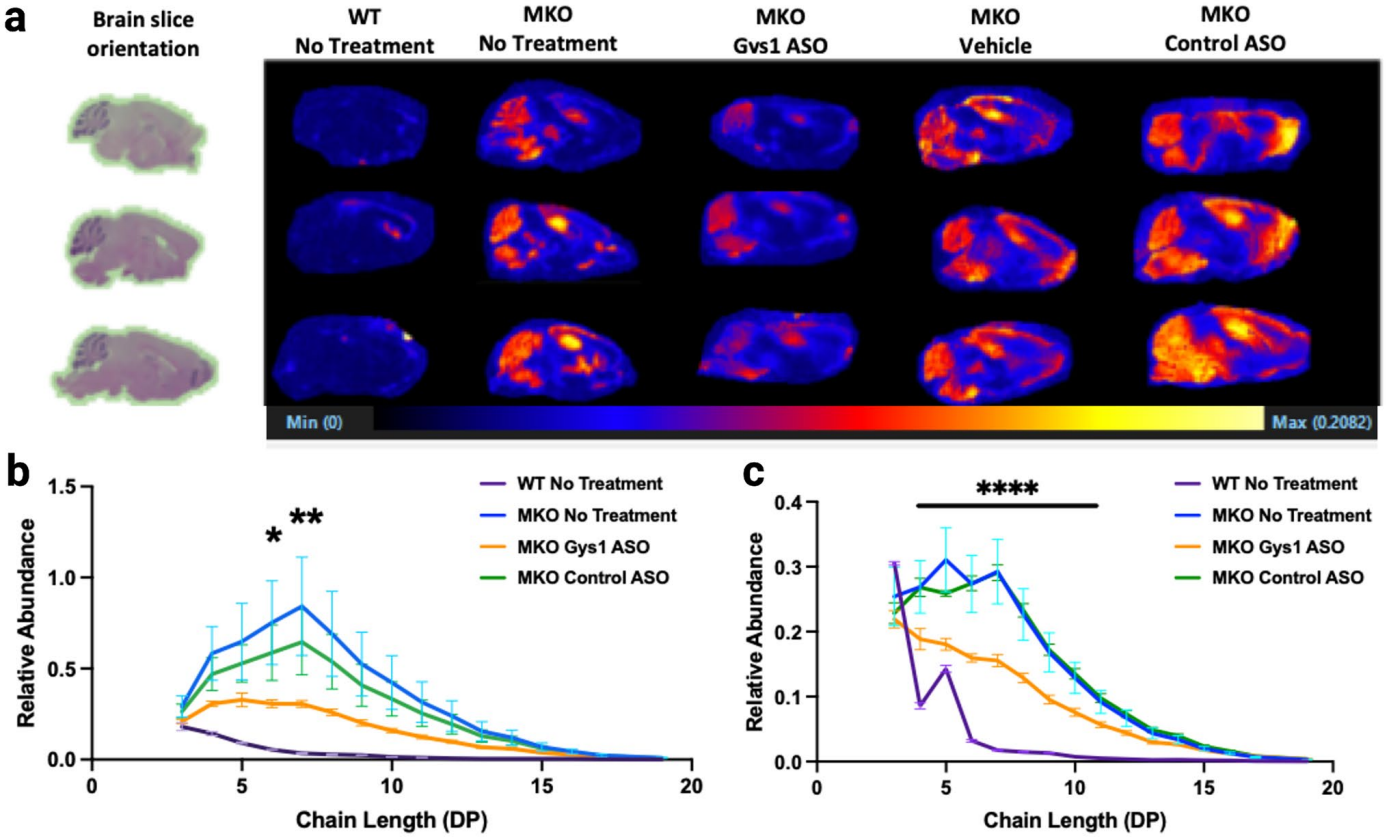
MALDI imaging of glycogen accumulation in mice. **a** Left, H&E staining of WT mouse brain reflecting the orientation for all the brains analyzed via MALDI. Right, MALDI imaging displaying regional and relative abundance of glycogen in WT and MKO mouse brain sagittal sections. Glycogen in this image was assessed by quantifying linear glucose chains that are 7 sugar monomers long. **b** Quantification of MALDI imaging in the hippocampus. The relative abundance of each glucose polymer from triose through DP19 (i.e. 19 glucose unit chain) was determined in the hippocampus from each cohort of mice (*N* = 3). The asterisks indicate there is a significant difference between the MKO Gys1 ASO-treated group and the MKO group with no treatment. **c** Similar analysis as **b** was also performed on the cerebellum (*N* = 3). Significance was determined using a one-way ANOVA with post hoc Tukey test for multiple comparisons between the MKO Gys1 ASO group and the MKO group with no treatment, where *P < 0.05, **P < 0.01, and ****P < 0.0001

A method utilizing matrix-assisted laser desorption/ionization (MALDI) coupled with mass spectrometry was recently developed to define the spatial glucose chain length distribution of glycogen [31]. In LD mouse models, glucose chain length is extended compared to wild-type glycogen, which promotes LB aggregation [10, 36]. Sagittal slices from paraffin blocks were treated with a citraconic acid buffer for antigen retrieval and then sprayed with an isoamylase matrix to hydrolyze glycogen at α-1,6-branch points to produce linear glucose chains. The treated slices then underwent MALDI coupled with time-of-flight detection to profile the quantity of these linear glucose chain distributions from glycogen among different brain regions, which can be used to quantify glycogen levels. The malin KO mice treated with Gys1-ASO displayed significantly less glycogen accumulation in all brain regions compared to the control groups, in agreement with the IHC staining (Fig. 4a). Quantification of the abundance of linear glucose chain lengths in both the hippocampal and cerebellum regions confirmed a reduction in glycogen levels for Gys1-ASO-treated mice compared to the control groups (Fig. 4b and c).

### Gys1-ASO Treatment Decreases Epileptiform Discharges in Malin KO Mice

**Fig. 5:**
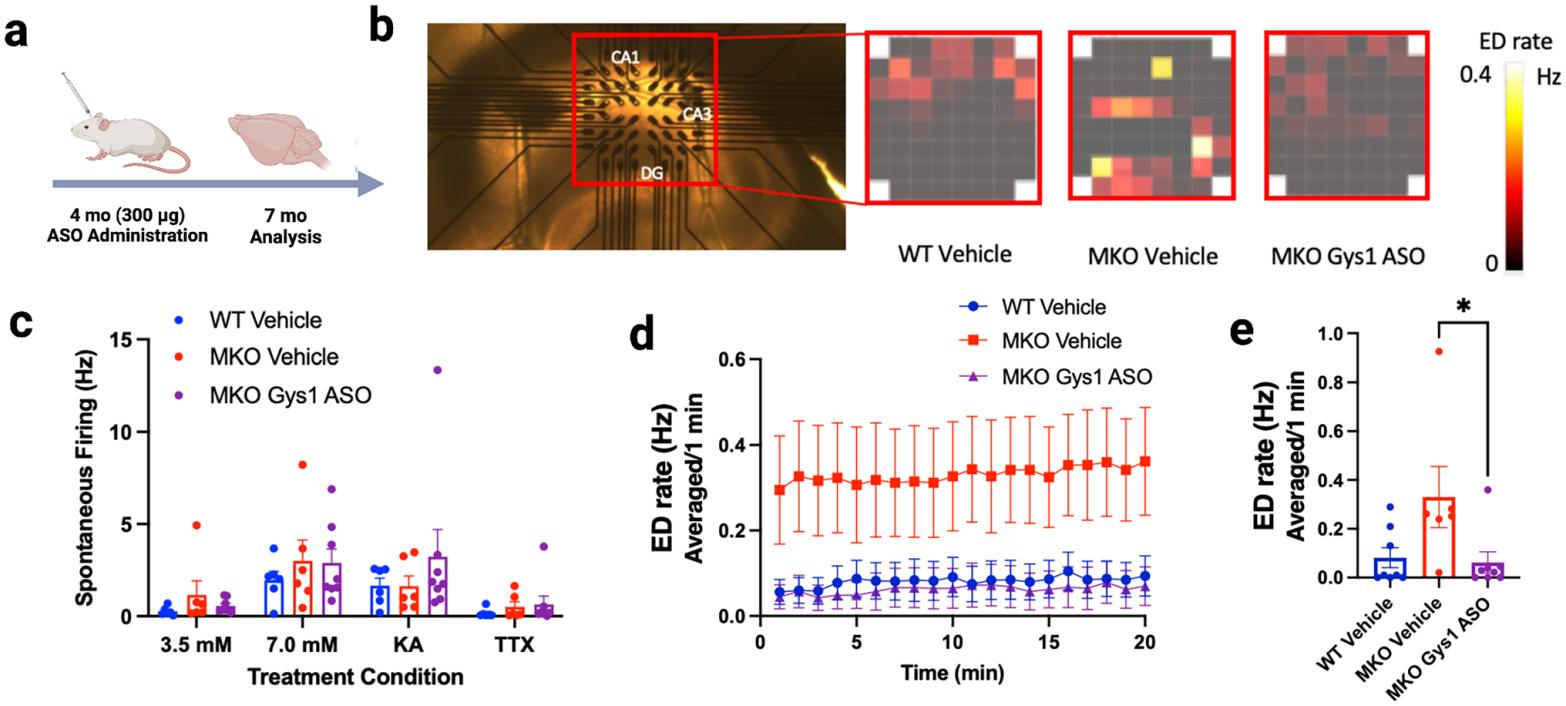
Electrophysiology analysis of the hippocampus using multi-electrode array. **a** Schematic showing treatment regimen for MEA experiments. Created with Biorender.com. **b** Representative picture of hippocampal slice on the electrode grid with regions CA1, CA3, and DG labelled. Red box call-outs show representative heatmap depicting spontaneous firing average from hippocampal slices in WT vehicle, MKO vehicle, and MKO Gys1-ASO-treated mice during the control period. **c** Average of 30-s mean firing rate and SEM for each validated electrode aggregated from each mouse for the last 5 min of each recording condition, significance determined using mixed effects ANOVA and Tukey’s post hoc comparisons. **d** Quantification of epileptiform discharge (ED) rate averaged over 1-min intervals for the 20-min control period. **e** Quantified mean ED rate and SEM averaged for each mouse in the different experimental groups. Significance between groups determined using an unpaired Student’s *t*-test. *P < 0.05

Multiple studies have demonstrated that LBs drive disease progression. Indeed, crossing LD mouse models with genetic models that reduce glycogen levels eliminates early-onset neurodegeneration and normalizes seizure susceptibility [21–26]. Recently, multiple groups have developed pre-clinical LD therapies that include enzyme therapy to degrade LBs and both Gys1-ASO and AAV-based therapies to down-regulate Gys1 [27, 37–41]. To date, no study has tested if treatment impacts epileptiform activity, which is a key hallmark of the disease. Since administration of the Gys1-ASO significantly reduced the progression of LB aggregation, we sought to test the impact on epileptiform discharges (ED). Four-month-old malin KO and wild-type littermates were administered either vehicle or Gys1-ASO via ICV injection. After 3 months, the mice were sacrificed, and slices from contralateral hippocampi were placed on electrodes for multi-electrode analysis (MEA) to record spontaneous firing (SF) and ED activity (Fig. 5a and b, Sup. Fig. 3). ED activity was defined as an epileptiform event with an amplitude greater than 15 µV, with a 200-ms deadtime after each detected event. The SF rate and number of ED events were averaged for each mouse and plotted. The SF rate was significantly modulated by recording conditions, but the SF did not significantly vary between experimental groups (Fig. 5c). For all mice, the SF significantly increased between the 3.5- and 7.0-mM K^+^ concentration conditions (*P* < 0.0001). The TTX significantly reduced SF from the 7.0-mM K^+^ concentration condition (*P* < 0.0003), confirming that SF was due to sodium spikes. These data indicate that the baseline firing rate did not vary between experimental groups. In contrast to SF, the malin KO vehicle-injected mice displayed higher rates of ED compared to the wild-type controls (Fig. 5d and e). Conversely, malin KO mice treated with Gys1-ASO exhibited a significant decrease in ED over the same time period when compared to the vehicle-injected malin KO mice (Fig. 5e).

## Discussion

LD impacts seemingly previously healthy teens with progressive epilepsy and childhood dementia. There is currently no treatment for this devastating disease, but targeting glycogen synthesis via Gys1-ASO administration is a promising pre-clinical strategy. ASO treatment is a safe and well-tolerated technique that has been utilized in pre-clinical settings for more than 50 years [42]. There are ten ASO drugs that have received FDA approval to date and more than 100 ASO drugs at multiple stages of clinical trials for a number of diseases [42, 43]. In this study, we demonstrate that the Gys1-ASO treatment is effective at decreasing glycogen synthase levels. The reduction in Gys1 protein levels corresponds to decreased glycogen accumulation and reduced ED in a malin KO model of LD, suggesting that slowing glycogen aggregation into LBs reduces neuronal excitability.

The mechanism of the Gys1-ASO functions by binding to *Gys1* mRNA at a target site that activates RNase H1, which accelerates the degradation rate of the target mRNA [27, 44]. Previous studies have demonstrated that this mechanism achieves broad distribution throughout all regions of the brain, with the highest ASO levels detected in the cortex and the lowest levels in the globus pallidus, caudate, and putamen [45]. While only a marginal decrease in *Gys1* mRNA was observed, the treatment with Gys1-ASO led to a significant decrease in Gys1 protein expression. This phenomenon has been observed in multiple studies, where a small change in *Gys1* mRNA results in a significant change in Gys1 protein expression [23, 37, 38]. If this phenomenon holds true in humans, then patients would only need to see a small decrease in mRNA levels to potentially experience significant knockdown of GYS1 activity and halt glycogen aggregation into LBs.

Aggregation and dysregulation of glycogen has been linked to multiple neurological diseases in recent years, including Pompe disease, amyotrophic lateral sclerosis, and Alzheimer’s disease [46–51]. Therefore, the ability to target and control glycogen synthesis in the brain is a potential therapeutic strategy for multiple diseases. Indeed, Maze Therapeutics recently demonstrated promising pre-clinical results for small molecule inhibition of Gys1 to treat Pompe disease in murine and canine models [52, 53]. Gys1 is responsible for driving glycogen synthesis in all tissues except the liver, which utilizes Gys2. While glycogen is an important energy source in many organs and plays a role in learning and hypoxia tolerance in the brain, multiple groups have demonstrated that an approximate 50% reduction in glycogen levels does not produce adverse health effects in mouse models [48, 54–58]. Indeed, Gys1-heterozygous animals do not exhibit abnormalities despite synthesizing only approximately 50% of wild-type levels of glycogen, and wild-type animals in our study that received the Gys1-ASO did not exhibit any obvious negative phenotypes [54]. Several studies with Gys1-heterozygous mice demonstrate that they maintain similar weight to their wild-type counterparts, normal levels of serum glucose and lactate, normal lifespan, and no significant change in locomotor activity, balance, or gait [54, 55]. Furthermore, recent case studies published on patients with glycogen storage disease 0, which is an autosomal recessive condition resulting from loss of function mutations in muscle glycogen synthase, reveal that parents with one null copy of GYS1 retain normal memory and learning capacity and are healthy, with adverse effects only noted in the offspring receiving two null copies of *GYS1* [59, 60].

In addition to studying the impact of decreased glycogen synthase activity, multiple studies have examined the impact of a mutation in another gene that leads to decreased glycogen synthesis in the skeletal muscle and heart [61, 62]. The *PPP1R3A* gene encodes a positive regulator of glycogen synthesis, but there is a truncating mutation of *PPP1R3A* that results in ~ 65% reduction of glycogen synthesis [61]. When this mutation was introduced into a mouse model, the same ~ 65% decrease in glycogen synthesis occurred and no cardiac or other disease phenotype was identified in the mice [61]. A recent study quantified the health outcomes in humans with this truncation mutation using UK Biobank data [62]. Their study reported ventricular ejection fraction, wall thickness, maximum heart rate, and maximum workload, finding no association between PPP1R3A truncation and cardiac defects. They also did not observe changes in serum metabolites or glucose levels [62]. These data strongly suggest that a 65% reduction in glycogen synthesis in skeletal muscle and heart is well tolerated. Similar to PPP1R3A, reduction of glycogen accumulation by Gys1-ASO was well-tolerated with no adverse symptoms in the current study. While the Gys1-ASO administered via ICV does not cross the blood–brain barrier, LD patients do accumulate LBs in muscle and heart tissue. Future studies could address the potential of utilizing this therapy to reduce LB accumulation in the peripheral nervous system [63].

The results with malin KO mice demonstrate similar efficacy in reducing LB levels as a previous study that utilized laforin KO mice and the same Gys1-ASO. Of the patients with LD, about half of them have a mutation in *EPM2A* while the other half have a mutation in *EPM2B* [16, 19, 64]. Patients who are screened for epilepsy genes and identify mutations in *EPM2A* or *EPM2B* could potentially receive this treatment to prevent glycogen accumulation and LD aggregation in the brain.

Equally important, the Gys1-ASO in this study achieved broad brain biodistribution and exhibits significant reduction of Gys1 throughout all brain regions. The Gys1 reduction resulted in decreased LB aggregates in all brain regions examined, including the hippocampus and cerebellum, where LBs are most prevalent in LD. Previous work demonstrated that an approximate 50% knockdown of Gys1 activity was sufficient to significantly reduce LB aggregation in murine models, and the data from this study supports that finding [21–23, 25].

Early screening could also be key for treatment efficacy given disease progression. Our studies confirmed previous results that MKO mice display higher levels of the neuroinflammatory markers CCL5, CXCL10, and MMP3 compared to WT mice [33]. In this study, the Gys1-ASO did not significantly decrease neuroinflammatory markers. This result is likely due to initiating treatment at 4 months of age in the MKO mice. Future work will define optimal timing for ASO treatment. Importantly, LD is a progressive disease where LD patients and LD mouse models develop seemingly normally, indicating that there is no primary epileptic circuit in the LD brain [4, 16, 17, 65]. In both LD mice and patients, LB formation precedes symptomatic disease [4, 66–68]. Initial symptoms arise in the teen years for patients and at 3–4 months of age in LD mice, while LBs are detectable in mice that are 8 weeks old [17, 24, 35, 69, 70]. Several laboratories utilizing multiple mouse models have demonstrated that LBs cause disease sequela, including myoclonus, perturbed synapse electrophysiology, increased susceptibility to seizure-inducing drugs, and progressive loss of both neuronal cells and cognitive function [16, 21–23, 25, 70, 71]. Current data suggest that early treatment that reduces total LB accumulation could ameliorate LB-driven disease.

While the specific regulation of glycogen by laforin and malin remains controversial, loss of function in either protein results in aberrant glycogen with increased phosphorylation and extended glucose chain lengths [24, 36, 72–74]. Previous studies have demonstrated that extended glucose chains form helices that decrease water solubility and increase glycogen aggregation to become LBs [9, 75]. Recent LB characterization revealed that this aberrant glycogen exhibits starch-like properties, with spontaneous formation of B-crystallinity, leading to insolubility and sequestration, forming LB aggregates [36]. Normally, there are two primary pathways for degrading glycogen in the brain: the brain isoform of glycogen phosphorylase (PYGB) is the primary enzyme responsible for degrading soluble glycogen in the brain, and alpha-glycosidase (GAA) is an enzyme expressed in the lysosome, responsible for degrading brain glycogen via glycophagy [48, 51, 76]. Neither of these degradation pathways successfully degrade or inhibit LBs in LD. As the aberrant glycogen aggregates into LBs, PYGB can no longer access the glucose moieties for degradation, and the rate of glycogen synthesis surpasses the ability of the glycophagy pathway to clear all the aggregates through normal GAA activity [41]. However, if one can reduce the rate of glycogen synthesis, then theoretically, these pathways could clear more of the aberrant glycogen before it aggregates into LBs. Our data, and previous work, support this hypothesis. Using multiple glycogen detection methods, we have demonstrated that treatment with Gys1-ASO significantly reduces glycogen accumulation throughout the brain in the malin KO LD mouse model. Combined with previous work from the Minassian laboratory showing similar results in the laforin KO LD model, we have demonstrated that the Gys1-ASO therapy successfully reduces LB accumulation regardless of the affected LD gene.

Another important hallmark of LD is epilepsy. Patients display progressive myoclonus epilepsy, experiencing seizures of increasing frequency and severity. In mouse models, mice display increased susceptibility to kainite-induced seizures. However, even before seizures are evident in patients, they display abnormal EEG readings with epileptiform activity. Both laforin KO and malin KO mouse models recapitulate this epileptiform activity as early as 3 months old [4, 77]. Importantly, the electrophysiology data suggests that the prevention of LB formation through Gys1-ASO administration reduces levels of epileptiform activity in the malin KO murine model. Multiple studies suggest a link between glycogen metabolism and neuronal excitability [78–80]. While the precise mechanism or mechanisms remain to be elucidated, several compelling hypotheses have been presented. Glycogen is an important storage molecule in the brain, not just for energy, but other critical substrates. Our recent work demonstrated that brain glycogen stores glucosamine, a critical sugar for protein glycosylation [32]. Glycosylation disorders have been linked to neuronal degradation and epilepsy [81]. Glycogenolysis is also linked with glutamine synthase activity. Glycogenolysis has been shown to promote K^+^ and glutamate uptake, and thereby glutamine synthesis, which play a critical role in neuronal excitability [78–80]. By reducing the rate of glycogen synthesis, it is possible that more glycogen remains accessible to degradation pathways, allowing glycosylation and glutamatergic neurotransmission to be less perturbed, thereby leading to a reduction in neuronal excitability and ED.

ASO treatment halts LB accumulation and reduces epileptiform discharges, but it does not appear to clear pre-existing LBs. However, other therapies are in development that degrade the LB aggregates. Extensive pre-clinical evaluation has been completed of antibody-enzyme fusions that employ antibody fragments as a delivery vehicle to transport enzymes capable of degrading LBs into the cytoplasm of neurons and astrocytes [39, 40, 81, 82]. This opens the possibility for a combination therapy, where existing LBs could be cleared with an antibody-enzyme fusion and then subsequent treatment with Gys1-ASO to prevent the formation of new LBs. Taken together, these therapies would have the potential to allow patients to live free from the devastating seizures and neurodegeneration driven by LB aggregation.

### Supplementary Information

Below is the link to the electronic supplementary material.

**Sup. Fig. 1: Quantification of Gys1 protein expression in mouse cohorts.** a) Additional Western blots of Gys1 protein expression in male mice. b) Western blots of Gys1 protein expression in female mice. c) Quantitation of Western blots for Gys1 protein expression in WT mouse cohorts. Statistical significance was calculated using an ordinary one-way ANOVA with post-hoc Tukey analysis for multiple comparisons, where * indicates P<0.05, ** P<0.01, *** P<0.001. (PNG 685 KB)

**Sup. Fig. 2: Quantification mRNA expression of inflammation markers in MKO mouse cohorts.** a) mRNA transcription levels of inflammation marker CCL5 in MKO and wildtype (WT) mouse cohorts b) mRNA transcription levels of inflammation marker CXCL10 in MKO and WT mouse cohorts c) mRNA transcription levels of inflammation marker MMP3 in MKO and WT mouse cohorts. All data were analyzed using one-way ANOVA with post-hoc Tukey test for multiple comparisons. No statistically significant differences in inflammation markers were found between MKO treatment groups or between WT treatment groups. However, all MKO mouse cohorts showed increased neuroinflammation markers compared to their WT counterparts. (PNG 287 KB)

**Sup. Fig. 3: Raw data sample from MEA experiments.** a) Picture of the hippocampal slice (from figure 5A) with raw data sample of firing activity recorded in a 7 mM K+ aCSF at the electrodes located within the red frame on the picture. b) raw data sample of ED occurring over the all the MEA electrode after perfusion of the slice in a 7 mM K+ aCSF. Data from 2 to 9 electrodes are averaged to quantify the ED rate. (PNG 944 KB)

## Data Availability

The data that support the findings of this study are available from the corresponding author, M.S.G., upon reasonable request.

## References

[1] Serratosa JM, Delgado-Escueta AV, Posada IJ, Shih S, Drury I, Berciano J (1995). The gene for progressive myoclonus epilepsy of the Lafora type maps to chromosome 6q. Hum Mol Genet.

[2] Serratosa JM, Gardiner RM, Lehesjoki AE, Pennacchio LA, Myers RM (1999). The molecular genetic bases of the progressive myoclonus epilepsies. Adv Neurol.

[3] Serratosa JM, Gómez-Garre P, Gallardo ME, Anta B, de Bernabé DB, Lindhout D (1999). A novel protein tyrosine phosphatase gene is mutated in progressive myoclonus epilepsy of the Lafora type (EPM2). Hum Mol Genet.

[4] Ganesh S, Delgado-Escueta AV, Sakamoto T, Avila MR, Machado-Salas J, Hoshii Y (2002). Targeted disruption of the Epm2a gene causes formation of Lafora inclusion bodies, neurodegeneration, ataxia, myoclonus epilepsy and impaired behavioral response in mice. Hum Mol Genet.

[5] Chan EM, Young EJ, Lanzano L, Munteanu I, Zhao X, Christopoulos CC (2003). Mutations in NHLRC1 cause progressive myoclonus epilepsy. Nat Genet.

[6] Gentry MS, Worby CA, Dixon JE (2005). Insights into Lafora disease: malin is an E3 ubiquitin ligase that ubiquitinates and promote the degradation of laforin. Proc Natl Acad Sci USA.

[7] Worby CA, Gentry MS, Dixon JE (2006). Laforin, a dual specificity phosphatase that dephosphorylates complex carbohydrates. J Biol Chem.

[8] Minassian BA, Lee JR, Herbrick JA, Huizenga J, Soder S, Mungall AJ (1998). Mutations in a gene encoding a novel protein tyrosine phosphatase cause progressive myoclonus epilepsy. Nat Genet.

[9] Sullivan AM, Nitschke S, Steup M, Minassian AB, Nitschke F (2017). Pathogenesis of Lafora disease: transition of soluble glycogen to insoluble polyglucosan. Intl J Mol Sci.

[10] Nitschke F, Sullivan MA, Wang P, Zhao X, Chown EE, Perri AM (2017). Abnormal glycogen chain length pattern, not hyperphosphorylation, is critical in Lafora disease. EMBO Mol Med.

[11] Nitschke F, Wang P, Schmieder P, Girard JM, Awrey DE, Wang T (2013). Hyperphosphorylation of glucosyl C6 carbons and altered structure of glycogen in the neurodegenerative epilepsy Lafora disease. Cell Metab.

[12] Tagliabracci VS, Turnbull J, Wang W, Girard JM, Zhao X, Skurat AV (2007). Laforin is a glycogen phosphatase, deficiency of which leads to elevated phosphorylation of glycogen in vivo. Proc Natl Acad Sci USA.

[13] Tagliabracci VS, Girard JM, Segvich D, Meyer C, Turnbull J, Zhao X (2008). Abnormal metabolism of glycogen phosphate as a cause for Lafora disease. J Biol Chem.

[14] Tagliabracci VS, Heiss C, Karthik C, Contreras CJ, Glushka J, Ishihara M (2011). Phosphate incorporation during glycogen synthesis and Lafora disease. Cell Metab.

[15] Mitra S, Gumusgoz E, Minassian BA (2022). Lafora disease: current biology and therapeutic approaches. Rev Neruol (Paris).

[16] Gentry MS, Guinovart JJ, Minassian BA, Roach PJ, Serratosa JM (2018). Lafora disease offers a unique window into neuronal glycogen metabolism. J Biol Chem.

[17] Turnbull J, Tiberia E, Striano P, Genton P, Carpenter S, Ackerley CA (2016). Lafora disease. Epileptic Disord.

[18] Nitschke F, Ahonen SJ, Nitschke S, Mitra S, Minassian BA (2018). Lafora disease - from pathogenesis to treatment strategies. Nat Rev Neurol.

[19] Pondrelli F, Muccioli L, Licchetta L, Mostacci B, Zenesini C, Tinuper P (2021). Natural history of Lafora disease: a prognostic systematic review and individual participant data meta-analysis. Orphanet J Rare Dis.

[20] Abubakr A, Wambacq I, Donahue JE, Zappulla R (2005). The presence of polyglucosan bodies in temporal lobe epilepsy: its role and significance. J Clin Neurosci.

[21] Duran J, Gruart A, Garcia-Rocha M, Delgado-Garcia JM, Guinovart JJ (2014). Glycogen accumulation underlies neurodegeneration and autophagy impairment in Lafora disease. Hum Mol Genet.

[22] Turnbull J, DePaoli-Roach AA, Zhao X, Cortez MA, Pencea N, Tiberia E (2011). PTG depletion removes Lafora bodies and rescues the fatal epilepsy of Lafora disease. PLoS Genet.

[23] Turnbull J, Epp JR, Goldsmith D, Zhao X, Pencea N, Wang P (2014). PTG protein depletion rescues malin-deficient Lafora disease in mouse. Ann Neurol.

[24] Duran J, Hervera A, Markussen KH, Varea O, López-Soldado I, Sun RC (2021). Astrocytic glycogen accumulation drives the pathophysiology of neurodegeneration in Lafora disease. Brain.

[25] Pederson BA, Turnbull J, Epp JR, Weaver SA, Zhao X, Pencea N (2013). Inhibiting glycogen synthesis prevents Lafora disease in a mouse model. Ann Neurol.

[26] Duran J, Tevy MF, Garcia-Rocha M, Calbó J, Milán M, Guinovart JJ (2012). Deleterious effects of neuronal accumulation of glycogen in flies and mice. EMBO Mol Med.

[27] Ahonen S, Nitschke S, Grossman TR, Kordasiewicz H, Wang P, Zhao X (2021). Gys1 antisense therapy rescues neuropathological bases of murine Lafora disease. Brain.

[28] Turnbull J, Wang P, Girard JM, Ruggieri A, Wang TJ, Draginov AG (2010). Glycogen hyperphosphorylation underlies Lafora body formation. Ann Neurol.

[29] Baba O (1993). Production of monoclonal antibody that recognizes glycogen and its application for immunohistochemistry. Kokubyo Gakkai Zasshi.

[30] Stanback AE, Conroy LR, Young LEA, Hawkinson TR, Markussen KH, Clarke HA (2021). Regional N-glycan and lipid analysis from tissues using MALDI-mass spectrometry imaging. STAR Protoc..

[31] Hawkinson TR, Sun RC (2022). Matrix-assisted laser desorption/ionization mass spectrometry imaging of glycogen in situ. Methods Mol Biol.

[32] Sun RC, Young LEA, Bruntz RC, Markussen KH, Zhou Z, Conroy LR (2021). Brain glycogen serves as a critical glucosamine cache required for protein glycosylation. Cell Metab.

[33] Lopez-Gonzalez I, Viana R, Sanz P, Ferrer I (2017). Inflammation in Lafora disease: evolution with disease progression in laforin and malin knock-out mice. Mol Neurobiol.

[34] Luna L (1986). AFIP manual of histologic staining methods.

[35] DePaoli-Roach AA, Tagliabracci VS, Segvich DM, Meyer CM, Irimia JM, Roach PJ (2010). Genetic depletion of the malin E3 ubiquitin ligase in mice leads to Lafora bodies and the accumulation of insoluble laforin. J Biol Chem.

[36] Brewer MK, Putaux JL, Rondon A, Uittenbogaard A, Sullivan MA, Gentry MS (2020). Polyglucosan body structure in Lafora disease. Carbohydr Polym.

[37] Gumusgoz E, Guisso DR, Kasiri S, Wu J, Dear M, Verhalen B (2021). Targeting Gys1 with AAV-SaCas9 decreases pathogenic polyglucosan bodies and neuroinflammation in adult polyglucosan body and Lafora disease mouse models. Neurotherapeutics.

[38] Gumusgoz E, Kasiri S, Guisso DR, Wu J, Dear M, Verhalen B (2022). AAV-mediated artificial miRNA reduces pathogenic polyglucosan bodies and neuroinflammation in adult polyglucosan body and Lafora disease mouse models. Neurotherapeutics.

[39] Brewer MK, Uittenbogaard A, Austin GL, Segvich DM, DePaoli-Roach A, Roach PJ (2019). Targeting pathogenic Lafora bodies in Lafora disease using an antibody-enzyme fusion. Cell Metab.

[40] Austin GL, Simmons ZR, Klier JE, Rondon A, Hodges BL, Shaffer R (2019). Central nervous system delivery and biodistribution analysis of an antibody-enzyme fusion for the treatment of Lafora disease. Mol Pharm.

[41] Gentry MS, Markussen KH, Donohue KJ (2022). Two diseases-one preclinical treatment targeting glycogen synthesis. Neurotherapeutics.

[42] Migliorati JM, Liu S, Liu A, Gogate A, Nair S, Bahal R (2022). Absorption, distribution, metabolism, and excretion of FDA-approved antisense oligonucleotide drugs. Drug Metab Dispos.

[43] Damase TR, Sukhovershin R, Boada C, Taraballi F, Pettigrew RI, Cooke JP (2021). The limitless future of RNA therapeutics. Front Bioeng Biotechnol.

[44] Crooke ST (2017). Molecular mechanisms of antisense oligonucleotides. Nucleic Acid Ther.

[45] Paymaan J, Powers B, Soriano A, Zhao H, Norris D, Matson J (2021). The atlas of RNase H antisense oligonucleotide distribution and activity in the CNS of rodents and non-human primates following central administration. Nucleic Acids Res.

[46] Mann DMA, Sumpter PQ, Davies CA, Yates PO (1987). Glycogen accumulations in the cerebral cortex in Alzheimer’s disease. Acta Neuropathol.

[47] Hicks J, Wartchow E, Mierau G (2011). Glycogen storage diseases: a brief review and update on clinical features, genetic abnormalities, pathologic features, and treatment. Ultrastruct Pathol.

[48] Duran J, Guinovart JJ (2015). Brain glycogen in health and disease. Mol Aspects Med.

[49] Tefera TW, Steyn FJ, Ngo ST, Borges K (2021). CNS glucose metabolism in amyotrophic lateral sclerosis: a therapeutic target?. Cell Biosci.

[50] Molares-Vila A, Corbalán-Rivas A, Carnero-Gregorio M, González-Cespón JL, Rodríguez-Cerdeira C (2021). Biomarkers in glycogen storage diseases: an update. Int J Mol Sci.

[51] Brewer MK, Gentry MS, DiNuzzo M, Schousboe A (2019). Brain glycogen structure and its associated proteins: past, present and future. Brain glycogen metabolism.

[52] Xi Y. Pharmacology of small molecule inhibitors of GYS1 in a mouse model of Pompe disease. Poster session presented at: WORLD Symposium; 2022; San Diego, CA.

[53] Choy R. Poster presentation: in-vitro characterization of MZE001, an orally active GYS1 inhibitor to treat Pompe disease. Poster session presented at: WORLD Symposium; 2022; San Diego, CA.

[54] Pederson BA, Chen H, Schroeder JM, Shou W, DePaoli-Roach AA, Roach PJ (2004). Abnormal cardiac development in the absence of heart glycogen. Mol Cell Biol.

[55] Pederson BA, Schroeder JM, Parker GE, Smith MW, DePaoli-Roach AA, Roach PJ (2005). Glucose metabolism in mice lacking muscle glycogen synthase. Diabetes.

[56] Chown EE, Wang P, Zhao X, Crowder JJ, Strober JW, Sullivan MA (2020). GYS1 or PPP1R3C deficiency rescues murine adult polyglucosan body disease. Ann Clin Transl Neurol.

[57] Lopez-Ramos JC, Duran J, Gruart A, Guinovart JJ, Delgado-Garcia JM (2015). Role of brain glycogen in the response to hypoxia and in susceptibility to epilepsy. Front Cell Neurosci.

[58] Duran J, Gruart A, Varea O, Lopez-Soldado I, Delgado-Garcia JM, Guinovart JJ (2019). Lack of neuronal glycogen impairs memory formation and learning-dependent synaptic plasticity in mice. Front Cell Neurosci.

[59] Sukigara S, Liang W, Komaki H, Fukuda T, Miyamato T, Saito T (2011). Muscle glycogen storage disease 0 presenting recurrent syncope with weakness and myalgia. Neuromuscul Disord.

[60] Kollberg G, Tulinius M, Gilljam T, Ostman-Smith I, Forsander G, Jotorp P (2007). Cardiomyopathy and exercise intolerance in muscle glycogen storage disease 0. N Engl J Med.

[61] Savage DB, Zhai L, Ravikumar B, Choi CS, Snaar JE, McGuire AC (2008). A prevalent variant in PPP1R3A impairs glycogen synthesis and reduces muscle glycogen content in humans and mice. PLoS Med.

[62] Homburger J. Genetic reduction of muscle glycogen is well tolerated in UK Biobank participants. Poster session presented at: WORLD Symposium; 2022; San Diego, CA.

[63] Benichou SA, Jauvin D, De Serres-Berard T, Pierre M, Ling KK, Bennett CF (2022). Antisense oligonucleotides as a potential treatment for brain deficits observed in myotonic dystrophy type 1. Gene Ther.

[64] Brewer MK, Machio-Castello M, Viana R, Wayne JL, Kuchtová A, Simmons ZR (2021). An empirical pipeline for personalized diagnosis of Lafora disease mutations. iScience..

[65] Raththagala M, Brewer MK, Parker MW, Sherwood AR, Wong BK, Hsu S (2015). Structural mechanism of laforin function in glycogen dephosphorylation and Lafora disease. Mol Cell.

[66] Baumann RJ, Kocoshis SA, Wilson D (1982). Lafora disease: liver histopathology in presymptomatic children. Ann Neurol.

[67] Ortolano S, Vieitez I, Agis-Balboa RC, Spuch C (2014). Loss of GABAergic cortical neurons underlies the neuropathology of Lafora disease. Mol Brain.

[68] Gomez-Garre P, Gutiérrez-Delicado E, Gómez-Abad C, Morales-Corraliza J, Villanueva VE, Rodríguez de Córdoba S (2007). Hepatic disease as the first manifestation of progressive myoclonus epilepsy of Lafora. Neurology.

[69] Lahuerta M, Gonzalez D, Aguado C, Fathinajafabadi A, García-Giménez JL, Moreno-Estellés M (2020). Reactive glia-derived neuroinflammation: a novel hallmark in Lafora progressive myoclonus epilepsy that progresses with age. Mol Neurobiol.

[70] Valles-Ortega J, Duran J, Garcia-Rocha M, Bosch C, Saez I, Pujadas L (2011). Neurodegeneration and functional impairments associated with glycogen synthase accumulation mouse model of Lafora disease. EMBO Mol Med.

[71] Garcia-Cabrero AM, Sánchez-Elexpuru G, Serratosa JM, Sánchez MP (2014). Enhanced sensitivity of laforin- and malin-deficient mice to the convulsant agent pentylenetetrazole. Front Neurosci.

[72] Worby CA, Gentry MS, Dixon JE (2008). Malin decreases glycogen accumulation by promoting the degradation of protein targeting to glycogen (PTG). J Biol Chem.

[73] Roach PJ (2015). Glycogen phosphorylation and Lafora disease. Mol Aspects Med.

[74] Sullivan MA, Nitschke S, Skwara EP, Wang P, Zhao X, Pan XS (2019). Skeletal muscle glycogen chain length correlates with insolubility in mouse models of polyglucosan-associated neurodegenerative diseases. Cell Rep.

[75] Koutsifeli P, Varma U, Daniels LJ, Annandale M, Li X, Neale JPH (2022). Glycogen-autophagy: molecular machinery and cellular mechanisms of glycophagy. J Biol Chem.

[76] Criado O, Aguado C, Gayarre J, Duran-Trio L, Garcia-Cabrero AM, Vernia S (2011). Lafora bodies and neurological defects in malin-deficient mice correlate with impaired autophagy. Hum Mol Genet.

[77] DiNuzzo M (2019). How glycogen sustains brain function: a plausible allosteric signaling pathway mediated by glucose phosphates. J Cereb Blood Flow Metab.

[78] Bak LK, Walls AB, Schousboe A, Waagepetersen HS (2018). Astrocytic glycogen metabolism in the healthy and diseased brain. J Biol Chem.

[79] Sickmann HM, Walls AB, Schousboe A, Bouman SD, Waagepetersen HS (2009). Functional significance of brain glycogen in sustaining glutamatergic neurotransmission. J Neurochem.

[80] Freeze HH, Eklund EA, Ng BG, Patterson MC (2012). Neurology of inherited glycosylation disorders. Lancet Neurol.

[81] Markussen KH, Macedo JKA, Machío M, Dolce A, Goldberg YP, Vander Kooi CW (2021). The 6th international Lafora epilepsy workshop: advances in the search for a cure. Epilepsy Behav.

[82] Gentry MS, Afawi Z, Armstrong DD, Delgado-Escueta A, Goldberg YP, Grossman TR (2020). The 5th international Lafora epilepsy workshop: basic science elucidating therapeutic options and preparing for therapies in the clinic. Epilepsy Behav.

