# Supplementary figures and images for: Gys1 Antisense Therapy Prevents Disease-Driving Aggregates and Epileptiform Discharges in a Lafora Disease Mouse Model
Source: Neurotherapeutics. 2023 Sep 12;20(6):1808–19. doi: 10.1007/s13311-023-01434-9 (PMC10684475; doi:10.1007/s13311-023-01434-9)

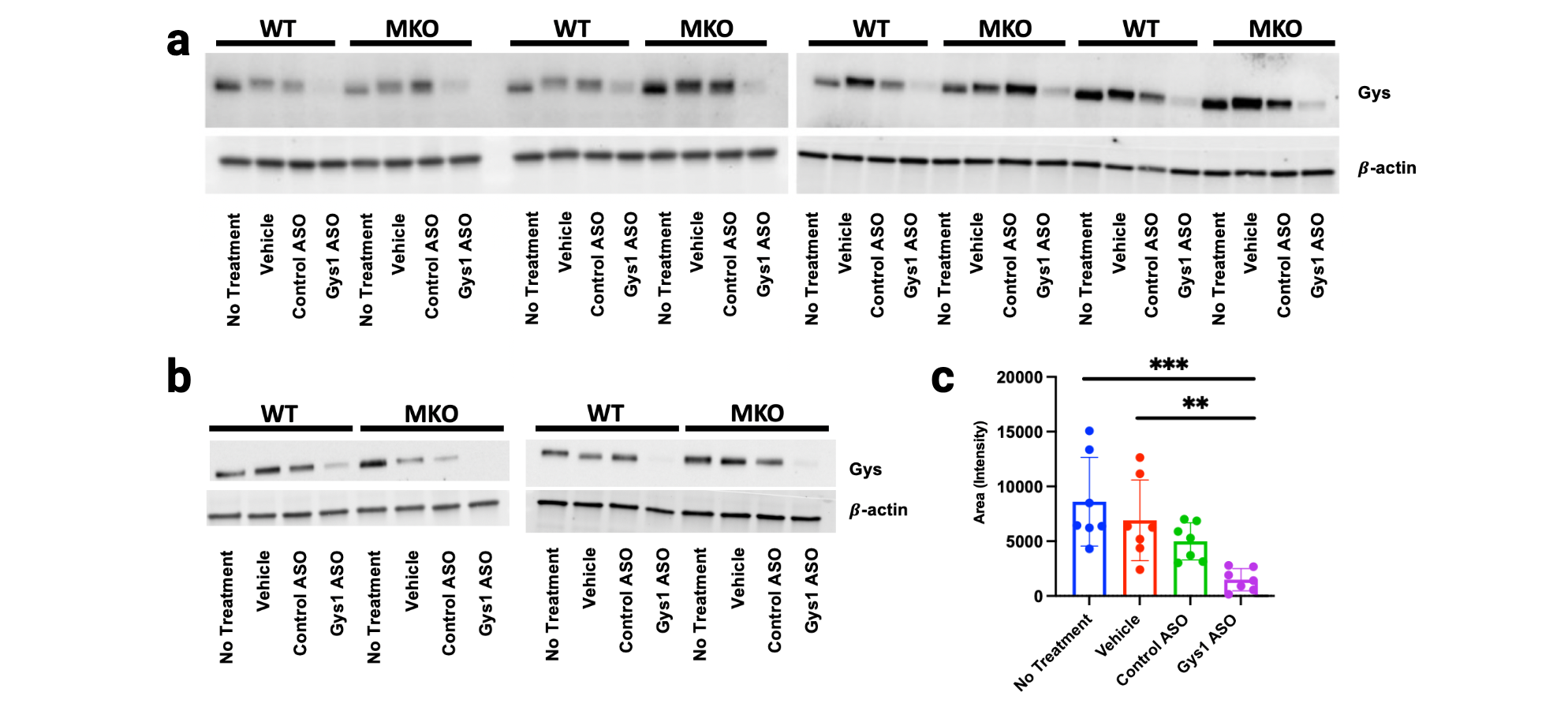

Supplement: Supplementary file 1 — Sup. Fig. 1: Quantification of Gys1 protein expression in mouse cohorts. a) Additional Western blots of Gys1 protein expression in male mice. b) Western blots of Gys1 protein expression in female mice. c) Quantitation of Western blots for Gys1 protein expression in WT mouse cohorts. Statistical significance was calculated using an ordinary one-way ANOVA with post-hoc Tukey analysis for multiple comparisons, where * indicates P<0.05, ** P<0.01, *** P<0.001. (PNG 685 KB) [file 13311_2023_1434_MOESM1_ESM.png]

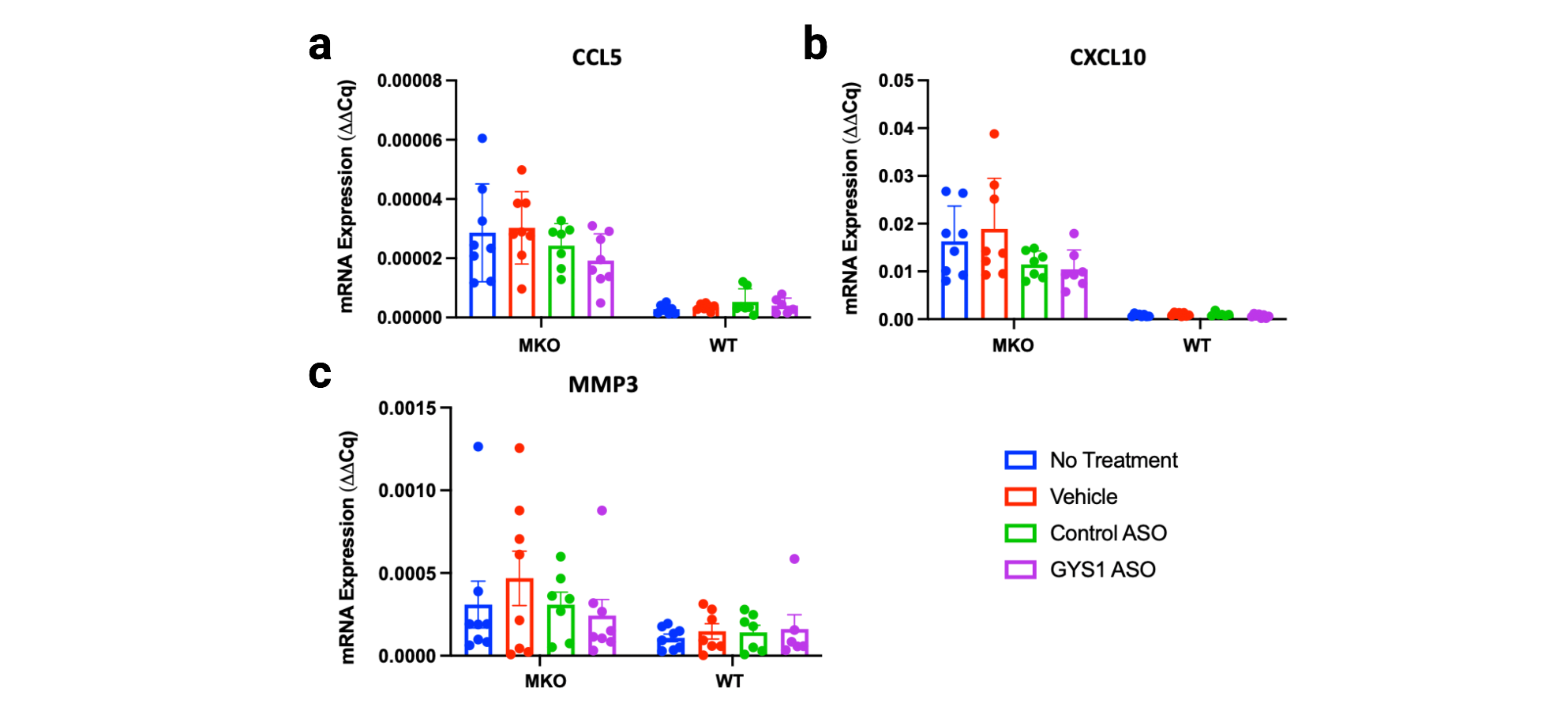

Supplement: Supplementary file 2 — Sup. Fig. 2: Quantification mRNA expression of inflammation markers in MKO mouse cohorts. a) mRNA transcription levels of inflammation marker CCL5 in MKO and wildtype (WT) mouse cohorts b) mRNA transcription levels of inflammation marker CXCL10 in MKO and WT mouse cohorts c) mRNA transcription levels of inflammation marker MMP3 in MKO and WT mouse cohorts. All data were analyzed using one-way ANOVA with post-hoc Tukey test for multiple comparisons. No statistically significant differences in inflammation markers were found between MKO treatment groups or between WT treatment groups. However, all MKO mouse cohorts showed increased neuroinflammation markers compared to their WT counterparts. (PNG 287 KB) [file 13311_2023_1434_MOESM2_ESM.png]

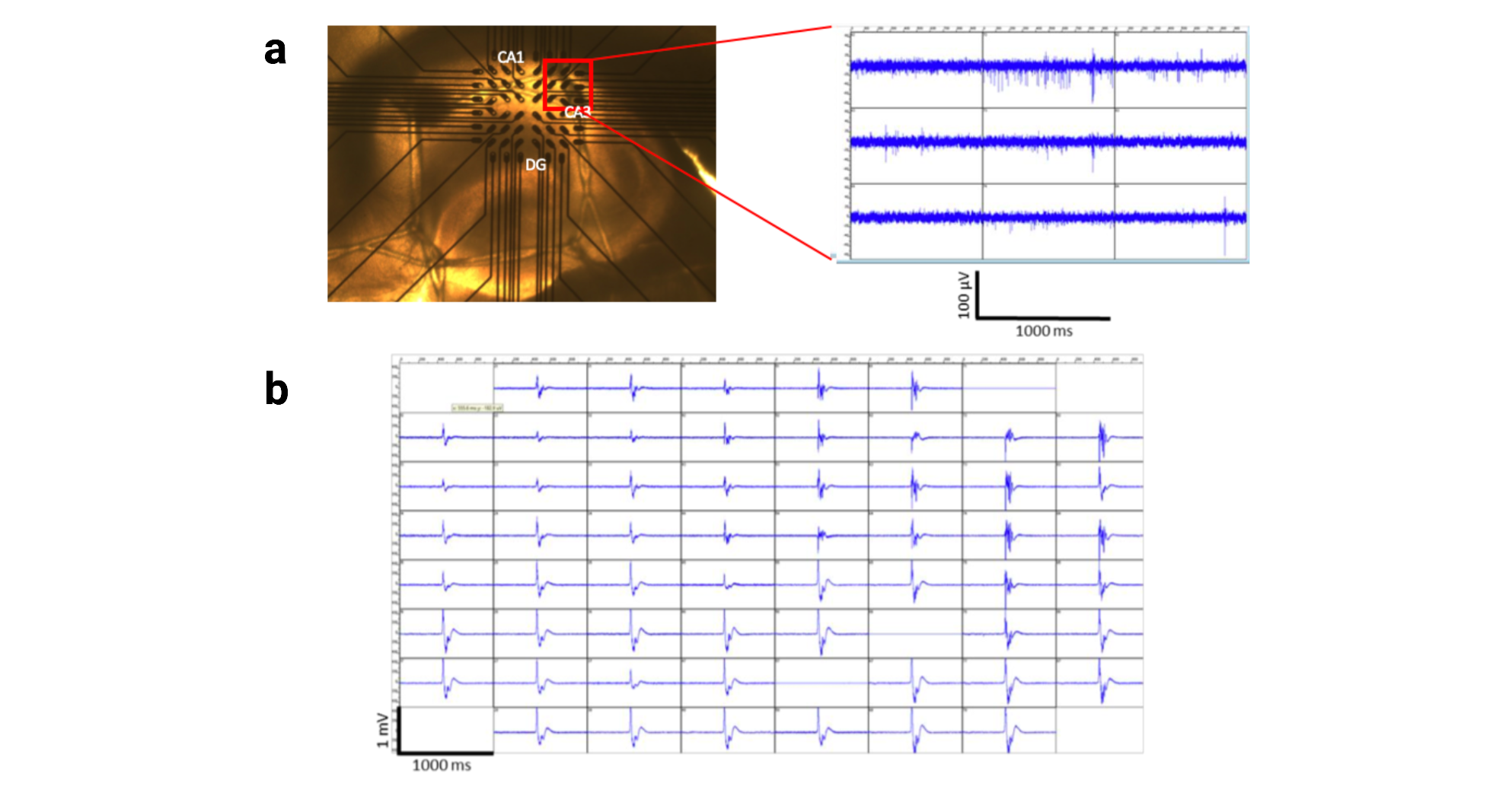

Supplement: Supplementary file 3 — Sup. Fig. 3: Raw data sample from MEA experiments. a) Picture of the hippocampal slice (from figure 5A) with raw data sample of firing activity recorded in a 7 mM K+ aCSF at the electrodes located within the red frame on the picture. b) raw data sample of ED occurring over the all the MEA electrode after perfusion of the slice in a 7 mM K+ aCSF. Data from 2 to 9 electrodes are averaged to quantify the ED rate. (PNG 944 KB) [file 13311_2023_1434_MOESM3_ESM.png]
